# Association between Mixing Ability of Masticatory Functions Measured Using Color-Changing Chewing Gum and Frailty among Japanese Older Adults: The Kyoto–Kameoka Study

**DOI:** 10.3390/ijerph17124555

**Published:** 2020-06-24

**Authors:** Daiki Watanabe, Tsukasa Yoshida, Keiichi Yokoyama, Yasuko Yoshinaka, Yuya Watanabe, Takeshi Kikutani, Mitsuyoshi Yoshida, Yosuke Yamada, Misaka Kimura, Kyoto-Kameoka Study Group

**Affiliations:** 1 National Institute of Health and Nutrition, National Institutes of Biomedical Innovation, Health and Nutrition, Tokyo 162-8636, Japan; t-yoshida@nibiohn.go.jp (T.Y.); yuwatana@mail.doshisha.ac.jp (Y.W.); yamaday@nibiohn.go.jp (Y.Y.); 2 Institute for Active Health, Kyoto University of Advanced Science, Kyoto 621-8555, Japan; physiol.yokoyama@gmail.com; 3 Laboratory of Applied Health Sciences, Kyoto Prefectural University of Medicine, Kyoto 602-8566, Japan; 4 Senior Citizen’s Welfare Section, Kameoka City Government, Kyoto 621-8501, Japan; 5 Center for Faculty Development, Kyoto University of Advanced Science, Kyoto 621-8555, Japan; yoshinaka.yasuko@kuas.ac.jp; 6 Faculty of Health and Sports Science, Doshisha University, Kyoto 610-0394, Japan; 7 Division of Rehabilitation for Speech and Swallowing Disorders, Nippon Dental University, Tokyo 184-0011, Japan; kikutani@tokyo.ndu.ac.jp; 8 Department of Advanced Prosthodontics, Hiroshima University Graduate School of Biomedical & Health Sciences, Hiroshima 739-0046, Japan; mitsu@hiroshima-u.ac.jp; 9 Department of Nursing, Doshisha Women’s College of Liberal Arts, Kyoto 610-0395, Japan

**Keywords:** mixing ability, color-changing chewing gum, frailty, older adults, cross-sectional study

## Abstract

The relationship between mixing ability of masticatory functions and frailty has not been well evaluated. This study investigated the prevalence of physical and comprehensive frailty and its association with mixing ability in 1106 older adults aged ≥65 years who underwent physical examination as part of the Japanese Kyoto–Kameoka Study. Mixing ability was assessed using color-changing chewing gum (1–5 points, 5 representing the best mixing ability). Participants were divided into four groups (5 points, 4 points, 3 points, and 1 or 2 points). The modified Japanese versions of the Cardiovascular Health Study (mJ-CHS) criteria and the validated Kihon Checklist (KCL) were used to assess physical and comprehensive frailty, respectively. Multivariate logistic regression was used to evaluate the association between frailty and mixing ability. The prevalence of physical and comprehensive frailty was 11.8% and 27.9%, respectively. After adjusting for confounders, the odds ratios of physical and comprehensive frailty comparing the highest to the lowest chewing gum score groups were 3.64 (95% confidence interval (CI): 1.62 to 8.18; *p* for trend = 0.001) and 2.09 (95% CI: 1.09 to 4.03; *p* for trend = 0.009), respectively. Mixing-ability tests involving chewing gum may be an indicator associated with both physical and comprehensive frailty.

## 1. Introduction

Frailty is a condition in which multiple physiological systems decline in function owing to a loss of homeostasis in response to stress [1,2] and is considered a worldwide public health problem among older adults [3]. Two major concepts of frailty exist in the literature: the Fried phenotype (FP) model, which defines frailty based on five criteria (physical frailty) [1,4,5], and the health deficits model (e.g., the Rockwood Frailty Index), which defines frailty as a multidimensional clinical geriatric syndrome (comprehensive frailty) [2,4,6,7]. The Kihon Checklist (KCL) is considered a valid tool for assessment of comprehensive frailty as a multidimensional clinical geriatric syndrome and is widely used in Japan and other countries [4,8,9,10]. Overall, frailty is associated with increased risk of death [8,11,12], disability [8,13], and burden of disease using disability-adjusted life years [14] in older adults. Accordingly, there is a need to establish objective, simple tools for the evaluation of frailty, with concepts rooted in both conceptual models.

The prevalence of oral disease among older adults is high, and the number of people with poor masticatory functions increases with age [15]. Decline in masticatory functions limits food choices and reduces the enjoyment of eating, making it difficult to secure sufficient food intake to maintain physiological function, which, in turn, leads to malnutrition [16,17]. An association between poor masticatory functions and frailty is thus likely [18,19]. In fact, a number of previous studies have reported that masticatory functions are positively associated with grip strength [20,21] and gait speed [20], indicating that it could be used to assess physical function in older adults.

Previous epidemiological studies have investigated masticatory functions with self-reported measures, primarily questionnaire-based [22], which have also assessed the number of natural teeth of participants and denture use [23]. Objective methods of evaluating masticatory functions, likely to provide more accurate results, often require specialist technology and skill [17]. Recent studies have confirmed the validity of mixing-ability assessment using color-changing chewing gum in terms of the number of teeth [24], tongue motor function, tongue pressure [25], and chew count [26] and found that gum chewing can objectively measure masticatory performance without the requirement for specialist technology or skill. Previous studies have reported that poor oral health including a lower mixing ability is associated with physical [18,19,24,27] and comprehensive frailty [28,29]. However, those previous studies have focused only on frailty, and the association of mixing ability with the subdomains of each assessment tool for frailty has not been well evaluated. In particular, although one subdomain of the comprehensive frailty defined by KCL includes oral function, sensitivity analyses that examine the relationship between mixing ability and each domain of frailty are also needed. This study aimed to investigate the association between objective mixing ability of masticatory functions and physical and comprehensive frailty using two validated frailty assessment tools in a community-based cohort study of older adults. In addition, we also examined the association between mixing ability and the subdomains of each assessment tool for frailty. Although the term “frailty” appears simple, the definition of frailty varies between studies [30]. Therefore, the association between mixing ability and frailty defined using a multiple assessment tool should be evaluated because there are many such tools in existence, with greater heterogeneity in their classification and predictive abilities.

## 2. Materials and Methods

### 2.1. Study Design and Population

The Kyoto–Kameoka Study is a cohort study of older residents of Kameoka City, Kyoto Prefecture, aged ≥65 years, which conducted the Needs in the Sphere of Daily Life survey (baseline survey), including the KCL, on 13,294 residents on July 29, 2011 [31,32,33,34,35]. The Health and Nutrition Status Survey (additional survey) was conducted as a follow-up to the original surveys, and 8319 residents submitted valid responses. For our study, we randomly selected 10 from 21 areas that make up Kameoka City and sent postcards to 4831 residents inviting them to undergo a physical checkup [31]. Of those residents, 1379 participants underwent a physical checkup examination as part of the Kyoto–Kameoka Study during March or April 2012. Written informed consent was obtained from all the participants before data acquisition. Health-related information, including medical history, socioeconomic status, smoking status and alcohol consumption, and physical activity, was extracted from the baseline and additional surveys.

The participants of the present study were respondents for whom baseline data were available (*n* = 1379), excluding those with incomplete responses to the modified Japanese version of the Cardiovascular Health Study (mJ-CHS) criteria (*n* = 75) or the KCL (*n* = 124) and those with missing color-changing chewing gum data (*n* = 74). Ultimately, 1106 participants were included in this study.

This study was approved by the ethics review boards of the Kyoto Prefectural University of Medicine (No. RBMR-E-363), Kyoto University of Advanced Science (No. 20-1), and the National Institute of Health and Nutrition (No. NIHN187-3). The study was carried out in accordance with the principles of the Declaration of Helsinki.

### 2.2. Frailty Definitions

We assessed physical and comprehensive frailty according to the mJ-CHS criteria and the KCL [30]. The J-CHS criteria, based on the CHS, modified for validity in Japanese individuals [9], are based on the FP model and include five elements of shrinking, exhaustion, low activity, slowness, and weakness. We evaluated physical frailty according to the mJ-CHS criteria, which are based on the J-CHS criteria with one question substituted for a question evaluating low activity: (I) Have you lost 2–3 kg or more in weight over the past 6 months without trying? If a participant responded “Yes” to this question, this was defined as “weight loss.” (II) Do you engage in sports, exercise, or keep-fit activities (other than walking?) If a participant responded “No” to this question, this was defined as “low activity.” (III) During the past two weeks, have you felt tired for no reason? If a participant responded “Yes” to this question, this was defined as “exhaustion.” (IV) If a participant’s grip strength was <26 kg for men and <18 kg for women, this was defined as “weakness.” (V) If a participant’s normal gait speed was <1.0 m/s, this was defined as “slow gait speed.” Individuals were defined as frailty if they met three out of five of the frailty reference criteria [9]. According to a prospective cohort study, FP-model-based physical frailty can also predict disability risk among older adults [36].

Grip strength was measured with a Smedley Hand Dynamometer (Grip-D TKK5101, Takei Scientific Instruments, Niigata, Japan). Measurements were taken twice for each hand, and the mean of the highest value for each hand was used. To evaluate each participant’s habitual gait speed, they were instructed to walk a 10-m distance at a comfortable pace. The time taken to walk a 6-m distance, excluding the first 2 m after acceleration and the final 2 m before deceleration, was measured with a digital stopwatch. Gait speed was calculated as distance divided by walking time.

The KCL is a self-administered questionnaire consisting of 25 questions in seven subdomains, covering instrumental activities of daily living (IADL), physical function, nutritional status, oral function, social status, cognitive status, and depression, which provide a comprehensive assessment of frailty. The KCL’s assessment principle thus resembles the health deficit model proposed by Rookwood et al. [6,7]. Every problem with activity or function received a point, and the higher the total score, the greater the difficulty in daily functioning, ranging from 0 (no frailty) to 25 (high frailty), with comprehensive frailty defined as a score of 7 or above [4,32,33,34,35]. The cutoff points of the KCL subdomains are the following: IADL disability, physical, nutrition, oral, social, cognitive, and depression defined as the IADL disability domain ≥10 points on 20 items including shopping; the physical inactivity domain ≥3 points on 5 items including walk continuously and history of fall; the malnutrition domain = 2 points on 2 items including physique; the oral dysfunction domain ≥2 points on 3 items including dry mouth and poor mastication; the socialization domain ≥1 point on 2 items including frequency of going out less; the memory domain ≥1 point on 3 items including memory loss; and the mood domain ≥2 points on 5 items including fulfilment and helpless [37]. In a prospective cohort study, KCL score was associated with increased risk of death or long-term care insurance certification within the next 3 years [8]. Additionally, we investigated the association between the subdomains evaluated using the mJ-CHS criteria and KCL [37] and mixing ability.

### 2.3. Measurement of Mixing Ability

Mixing ability was assessed using color-changing chewing gum (Xylitol Masticatory Performance Evaluating Gum, Lotte, Tokyo, Japan) [17,24,25,26,28,29,38,39]. This chewing gum contained 7 kcal energy, 2.3 g carbohydrate (0 g sugar), and 1.7 g xylitol (70 mm × 20 mm × 1 mm, 3.0 g). It contained ingredients that did not adhere to teeth or dentures as well as red, yellow, and blue dyes that change color when mixed with saliva and chewed. The red dyes are sensitive to pH changes, turning red under neutral or alkaline conditions. The unchewed gum contains citric acid, which maintains it in an acid environment (low pH) and is yellowish-green in color. When chewed and mixed with saliva, the internal pH of the gum changes from acidic to neutral or alkaline, turning it red. Therefore, a higher number of chews was associated with red colored gum, which indicated better mixing ability. This test principally evaluates the mixing ability of masticatory functions.

The study staff provided participants with detailed information on the chewing gum and explained the experimental protocol, instructing all participants to chew the gum as many times as possible in a 60-s period. They told participants to spit out the chewed gum into a piece of white tissue paper as soon as the measurement period was over and immediately compared it with the color chart on the packaging. If one of the study staff was unsure how to classify the color of the gum, they discussed it with other study staff before determining the color and assessing mixing ability. If the chewed gum contained more than one color (had not been thoroughly chewed), the study staff reduced the mixing ability score by 1 point. Mixing ability measured using color-changing chewing gum was graded in a range from 1 point (yellowish-green; the poorest mixing ability) to 5 points (red; the best mixing ability). Visual determination of chewing gum color was previously validated by comparison with chromaticity of colorimetry measurements [39].

### 2.4. Statistical Analysis

Given the small number of participants who scored 1 or 2 points, mixing ability measured using color-changing chewing gum was categorized into four groups: 5 points, *n* = 320; 4 points, *n* = 563; 3 points, *n* = 163; and 1 + 2 points, *n* = 60. Descriptive statistics representing continuous variables were expressed as mean (standard deviation), and groups were compared by analysis of variance (ANOVA). Categorical variables were expressed as counts and percentages, and between-group differences were assessed using the χ^2^ test. Missing values for covariates were supplemented with values from five data sets created by multiple imputation to perform multivariate imputation by chained equation (MICE) [40]. All missing values were assumed to be missing at random.

The prevalence of frailty in the four color-changing chewing gum score groups was measured as a case count with corresponding percentages. To adjust for potential confounders, we used multivariate logistic analysis including a number of baseline covariates. Multivariate analysis was performed using the following three models: Model 1 was adjusted for age, sex, and geographical area. Model 2 comprised covariates from model 1 with the addition of variables considered related to frailty, including body mass index (BMI), family structure, economic status, number of medications prescribed, history of hypertension, cerebral stroke, heart disease and dyslipidemia, alcohol intake, smoking, and physical activity. These covariates were selected based on the literature [22,32,33,34]. Model 3 comprised variables from model 2 with the addition of oral health-related variables such as use of dentures and daily teeth brushing. The results of these analyses were expressed as odds ratios (ORs) and 95% confidence intervals (95% CIs), with the ORs calculated with reference to the group with the best mixing ability. Additionally, we also examined the relationship between the subdomains of the mJ-CHS criteria and the KCL and mixing ability in a similar manner.

A two-sided *p* value < 0.05 was considered significant. All analyses were conducted using JMP Pro for Windows (SAS Institute, Inc., Cary, NC, USA) or R software 3.4.3 (R Core Team, Vienna, Austria).

## 3. Results

Participants with the lowest color-changing chewing gum score (the worst mixing ability) were older and had a higher rate of denture use (Table 1). This group also had a lower mean BMI, included fewer alcohol drinkers and people who brushed their teeth daily, had lower mean grip strength, and slower mean gait speed. 

The prevalence of both physical and comprehensive frailty was higher in groups with lower color-changing chewing gum scores, where it was 11.8% and 27.9%, respectively (Table 2). After adjusting for confounders, the ORs of physical and comprehensive frailty comparing the highest to the lowest chewing gum scores groups were 3.64 (95% CI: 1.62 to 8.18; *p* for trend = 0.001) and 2.09 (95% CI: 1.09 to 4.03; *p* for trend = 0.009), respectively. Groups with lower color-changing chewing gum scores had a higher prevalence of slow gait speed, weakness, exhaustion, low activity, and weight loss (Table 3). Even after adjusting for confounding factors, groups with lower color-changing chewing gum scores had a higher prevalence of weakness (weak grip strength), although there was no association with other factors. In addition, we demonstrated that groups with lower color-changing chewing gum scores had higher prevalence of oral frailty and depression, evaluated using the KCL subdomains (Table 4).

## 4. Discussion

In this study, we investigated the association between the mixing ability of masticatory functions and the prevalence of frailty in a population-based cohort of older adults. Even after adjusting for confounding factors, we found that mixing ability was associated with physical and comprehensive frailty. To our knowledge, this is the first study to demonstrate an association between measured mixing ability using color-changing chewing gum, the prevalence of physical and comprehensive frailty, and each domain of frailty. These findings suggest that objectively measured mixing ability may be an indicator associated with both physical and comprehensive frailty.

We evaluated physical frailty using the mJ-CHS criteria based on the proposed phenotype model by Fried et al. and comprehensive frailty using the KCL based on the proposed health deficit model by Rockwood et al. In this study, the prevalence of physical and comprehensive frailty was 11.8% and 27.9%, respectively. In a previous study, the reported prevalence of physical frailty assessed according to the J-CHS criteria in 16,251 community-dwelling older Japanese adults (mean age 75.1 years) was 11.2% [9]. We have previously reported that the prevalence of comprehensive frailty assessed according to the KCL (baseline survey) in a population of 13,294 older adults (mean age 74.5 years), from which our study group was drawn, was 30.8% for men and 33.3% for women [35]. There was thus no significant difference between physical and comprehensive frailty prevalence in our study population (mean age 73.4 years) and the populations investigated in larger studies. Accordingly, as our results reflect similar prevalence of frailty to that reported in previous studies, comparisons with these studies involve little bias and are therefore valid. In addition, we evaluated physical frailty according to the mJ-CHS criteria with one substitute question to evaluate low activity because we did not ask the participants the same question items as in the J-CHS criteria, but these differences do not seem to be a problem.

In this study, we also investigated the associations between mixing ability and physical and comprehensive frailty assessed using two different models. We found that objective mixing ability was associated with the prevalence of FP-model-based physical frailty, defined with parameters such as grip strength and gait speed. A decline in masticatory functions could be associated with physical performance because it limits food choices and reduces the enjoyment of eating, making it difficult to secure sufficient food intake to maintain physiological function and leading to malnutrition [16,17]. In a study of older adults, the mixing ability evaluated using color-changing chewing gum was associated with physical frailty [24] and sarcopenia [38], and these previous studies support our results. This suggests that mixing ability might be a surrogate marker reflecting the physical performance of older adults. Although further research is needed to understand underlying mechanisms and to establish the causal relationships on associations between mixing ability and frailty, markers of poor oral health could be useful indicators of frailty and valuable additions to health-screening assessments used in older people [27].

In addition, our results showed that, even after adjustment for oral health-related variables, e.g., denture use and daily teeth brushing, objectively measured mixing ability was associated with the prevalence of physical frailty as well as multifaceted comprehensive frailty, including in psychological, cognitive, social, and other domains. Previous prospective [28] and cross-sectional studies [29] have reported that a lower mixing ability is associated with comprehensive frailty, and these previous studies support our results. However, those previous studies have focused only on comprehensive frailty, and the association of mixing ability with the subdomains of assessment tool for frailty has not been well evaluated. Previous studies have reported that a lower masticatory ability is associated with higher depression and anxiety scores [17,41]. Our results indicate that the prevalence of depression defined using the KCL subdomain was higher in lower mixing ability individuals, and these previous studies support our results. Previous studies comparing comprehensive frailty with physical frailty have found that comprehensive frailty is a more accurate predictor of the risk of death [11,12]. This is because the index of comprehensive frailty is suggested to exhibit a positive linear correlation with age, reflecting biological aging [42]. Nevertheless, physical frailty is reportedly associated with increased risk of death and disability [43]. These findings suggest that assessing frailty in terms of multiple aspects may enable more accurate identification of high-risk groups. Our results suggest that measured mixing ability, using color-changing chewing gum, may be a useful tool for identifying groups at high risk of death or disability due to frailty.

One of the strengths of this study was that prevalence of frailty was assessed using two different validated methods based on established models of frailty [5,6,7]. Because we were able to investigate the association between objective mixing ability and the prevalence of frailty in a larger cohort of community-dwelling older adults compared with those of previous studies [24,29], our results may also be more widely generalizable.

However, this study has several methodological limitations. First, this was a cross-sectional study. Poor oral health is associated with numerous chronic diseases in addition to frailty, and oral diseases, such as periodontitis and tooth loss, are associated with social factors and lifestyle habits [44]. This means that neither a temporal nor a direct causal relationship can be inferred as underlying our observed association between mixing ability of masticatory functions and the prevalence of frailty. Second, although we selected our study participants from among Kameoka City residents by cluster random sampling, only 28.5% underwent a physical checkup examination. These participants may thus have been more health-aware than the general population of older adults, opening our study to the possibility of selection bias. Third, our study included participants with a history of diabetes, dyslipidemia, heart disease, and stroke. These limitations may interfere with the generalization of the results. However, our results were similar after excluding participants with these diseases. Finally, systematic error because of self-reporting could not be completely excluded from the results. Self-reported data in areas such as education, income, smoking, and medical history may thus have been affected by recall bias. In addition, although previous study has reported the reliability and validity of a quantitative color scale to evaluate mixing ability using color-changing chewing gum [39], we could not evaluate the inter-rater variability. The chewing gum color may be influenced by both masticatory performance and saliva amount. Although we took numerous implicit confounding factors into account, bias because of unmeasured confounding factors associated with masticatory performance and saliva amount could not be completely eliminated. However, as we did use multivariate analysis to adjust our results for known associated factors such as social and economic status, we were able to minimize the effect of confounders. Our results thus suggested that color-changing chewing gum might be an indicator associated with both physical and comprehensive frailty.

Considering the increasing interest in identifying frailty older people, color-changing chewing gum has an objective, easy, and solid methodology to evaluate simultaneously physical and comprehensive frailty suitable for the assessment of frailty, although the definition of frailty varies between studies [30]. If a self-administered questionnaire is used to assess physical and comprehensive frailty, participants may need be asked to answer the many questions contained in the questionnaires. Moreover, the questionnaire may be affected by self-reporting bias and cognitive function. Therefore, color-changing chewing gum can be used for the objective measurement of mixing ability without the need for specialist technology or skill and may thus be a useful early indicator of frailty in older adults.

## 5. Conclusions

The mixing ability of older adults as objectively measured using color-changing chewing gum was associated with the prevalence of both physical and comprehensive frailty. Given the rapidly increasing prevalence of frailty worldwide, its early discovery is important both for enabling people to stay healthy in old age and for limiting the burden of healthcare-related costs. Color-changing chewing gum may be useful indicators associated with both physical and comprehensive frailty in clinical practice and public health research.

## Figures and Tables

**Table 1:** Baseline characteristics of the study participants according to the color-changing chewing gum score ^a^.

	Color-Changing Chewing Gum Score								*p*-Value
	5 (*n* = 320)		4 (*n* = 563)		3 (*n* = 163)		1 + 2 (*n* = 60)		
Age (years) ^b^	72.2	(5.2)	73.2	(5.1)	75.1	(5.6)	76.7	(5.9)	**<0.001**
Women (*n* (%)) ^c^	152	(47.5)	282	(50.1)	94	(57.7)	30	(50.0)	0.208
BMI (kg/m^2^) ^b^	23.4	(2.9)	22.8	(3.0)	22.8	(2.8)	22.2	(3.6)	**0.011**
Alcohol drinker (*n* (%)) ^c^	237	(74.1)	386	(68.6)	107	(65.6)	34	(56.7)	**0.029**
Current smoker (*n* (%)) ^c^	26	(8.1)	48	(8.5)	7	(4.3)	5	(8.3)	0.350
MVPA (*n* (%)) ^c^	164	(51.3)	311	(55.2)	80	(49.1)	28	(46.7)	0.326
Grip strength (kg) ^b^	29.1	(8.7)	27.7	(7.6)	25.5	(8.2)	24.0	(7.8)	**<0.001**
Gait speed (m/s) ^b^	1.3	(0.2)	1.3	(0.2)	1.2	(0.2)	1.1	(0.2)	**<0.001**
Living alone (*n* (%)) ^c^	34	(10.6)	49	(8.7)	22	(13.5)	7	(11.7)	0.317
HSE (*n* (%)) ^c^	124	(38.8)	221	(39.3)	49	(30.1)	19	(31.7)	0.129
Education ≥13 y (*n* (%)) ^c^	91	(28.4)	153	(27.2)	30	(18.4)	16	(26.7)	**0.049**
Denture use (*n* (%)) ^c^	159	(49.7)	330	(58.6)	118	(72.4)	43	(71.7)	**<0.001**
Daily teeth brushing (*n* (%)) ^c^	311	(97.2)	525	(93.3)	151	(92.6)	46	(76.7)	**<0.001**
No medication (*n* (%)) ^c^	74	(23.1)	114	(20.3)	21	(12.9)	14	(23.3)	0.058
Hypertension (*n* (%)) ^c^	130	(40.6)	238	(42.3)	68	(41.7)	15	(25.0)	0.080
Stroke (*n* (%)) ^c^	6	(1.9)	17	(3.0)	5	(3.1)	5	(8.3)	0.063
Heart disease (*n* (%)) ^c^	29	(9.1)	65	(11.6)	20	(12.3)	5	(8.3)	0.557
Diabetes (*n* (%)) ^c^	26	(8.1)	62	(11.0)	14	(8.6)	5	(8.3)	0.498
Hyperlipidemia (*n* (%)) ^c^	39	(12.2)	72	(12.8)	20	(12.3)	7	(11.7)	0.990

Abbreviations: BMI = body mass index; HSE = high socioeconomic status; MVPA = moderate to vigorous physical activity; ^a^ Bolded *p*-values were statistically significant (*p* < 0.05). Participants with missing data underwent multiple imputation: alcohol status (*n* = 7); smoking status (*n* = 12); physical activity (*n* = 66); family structure (*n* = 41); socioeconomic status (*n* = 37); education attainment (*n* = 82); denture use (*n* = 12); brush teeth everyday (*n* = 4); and medications (*n* = 48). ^b^ Continuous variables are presented as mean (standard deviation) and were examined using the analysis of variance (ANOVA). ^c^ Categorical variables are presented as counts (%) and were examined using the χ^2^ test.

**Table 2:** Multivariable adjusted odds ratios and 95% confidence intervals of the prevalence of physical and comprehensive frailty according to the color-changing chewing gum score ^a^.

	Color-Changing Chewing Gum Score								*p* for Trend ^b^
	5 (*n* = 320)		4 (*n* = 563)		3 (*n* = 163)		1 + 2 (*n* = 60)		
**Physical**									
Case (*n* (%))	22	(6.9)	60	(10.7)	29	(17.8)	19	(31.7)	
Model 1 ^c^	1.00	(Ref)	1.44	(0.85 to 2.44)	2.01	(1.08 to 3.73)	3.97	(1.90 to 8.29)	**<0.001**
Model 2 ^d^	1.00	(Ref)	1.47	(0.85 to 2.55)	1.95	(1.02 to 3.72)	3.96	(1.79 to 8.74)	**<0.001**
Model 3 ^e^	1.00	(Ref)	1.44	(0.83 to 2.51)	1.94	(1.01 to 3.74)	3.64	(1.62 to 8.18)	**0.001**
**Comprehensive**									
Case (*n* (%))	65	(20.3)	154	(27.4)	61	(37.4)	29	(48.3)	
Model 1 ^c^	1.00	(Ref)	1.40	(1.00 to 1.98)	1.86	(1.19 to 2.89)	2.52	(1.37 to 4.61)	**<0.001**
Model 2 ^d^	1.00	(Ref)	1.43	(1.00 to 2.06)	1.70	(1.07 to 2.70)	2.27	(1.19 to 4.33)	**<0.001**
Model 3 ^e^	1.00	(Ref)	1.39	(0.97 to 2.01)	1.65	(1.03 to 2.64)	2.09	(1.09 to 4.03)	**0.009**

Abbreviation: ^a^ All values were counts (%) or relative odds ratio (95% confidence interval). All estimates are derived from multivariable logistic regression models. Fried phenotype model-based physical frailty is defined according to the Japanese version of the Cardiovascular Health Study criteria. The Kihon Checklist-based comprehensive frailty is defined as a score of 7 or above; ^b^ Linear trend *p*-values are calculated with the likelihood ratio test using ordinal variable (color-changing chewing gum score) values. Bolded *p*-values are statistically significant (*p* < 0.05); ^c^ Model 1 was adjusted for age (continuous), sex (woman or man), and population density (≥1000 or <1000 people/km^2^); ^d^ Model 2 included variables from model 1 as well as adjustment for BMI (continuous), physical activity (yes or no), smoking status (never smoker, past smoker, or current smoker), alcohol status (drinker or nondrinker), educational attainment (<9, 10–12, or ≥13 years), medications prescribed (continuous), living alone (yes or no), socioeconomic status (high or low), and history of disease (hypertension, diabetes, dyslipidemia, heart disease, and stroke; yes or no); ^e^ Model 3 included variables from model 2 plus adjustment for denture use (yes or no) and daily teeth brushing (yes or no).

**Table 3:** Multivariable adjusted odds ratios and 95% confidence intervals of the prevalence of each component of the modified Japanese versions of the Cardiovascular Health Study criteria according to the score of color-changing chewing gum ^a^.

	Color-Changing Chewing Gum Score								*p* for Trend ^b^
	5 (*n* = 320)		4 (*n* = 563)		3 (*n* = 163)		1 + 2 (*n* = 60)		
**Slow gait speed**									
Case (*n* (%))	31	(9.7)	53	(9.4)	22	(13.5)	13	(21.7)	
Model 1 ^c^	1.00	(Ref)	0.86	(0.53 to 1.41)	0.94	(0.50 to 1.76)	1.41	(0.64 to 3.07)	0.571
Model 2 ^d^	1.00	(Ref)	0.84	(0.50 to 1.39)	0.88	(0.46 to 1.68)	1.23	(0.54 to 2.80)	0.846
Model 3 ^e^	1.00	(Ref)	0.86	(0.52 to 1.44)	0.91	(0.47 to 1.76)	1.35	(0.58 to 3.14)	0.691
**Weakness**									
Case (*n* (%))	20	(6.3)	49	(8.7)	28	(17.2)	20	(33.3)	
Model 1 ^c^	1.00	(Ref)	1.20	(0.68 to 2.11)	1.91	(1.00 to 3.64)	4.37	(2.04 to 9.38)	**<0.001**
Model 2 ^d^	1.00	(Ref)	1.20	(0.66 to 2.16)	1.73	(0.88 to 3.38)	4.10	(1.82 to 9.27)	**<0.001**
Model 3 ^e^	1.00	(Ref)	1.14	(0.63 to 2.07)	1.66	(0.84 to 3.30)	3.59	(1.57 to 8.21)	**0.002**
**Exhaustion**									
Case (*n* (%))	81	(25.2)	144	(25.6)	59	(36.0)	22	(36.7)	
Model 1 ^c^	1.00	(Ref)	0.99	(0.72 to 2.41)	1.41	(0.92 to 2.16)	1.32	(0.72 to 1.37)	0.129
Model 2 ^d^	1.00	(Ref)	0.96	(0.69 to 1.34)	1.31	(0.84 to 2.03)	1.14	(0.61 to 2.14)	0.327
Model 3 ^e^	1.00	(Ref)	0.96	(0.69 to 1.35)	1.33	(0.85 to 2.08)	1.12	(0.59 to 2.12)	0.329
**Low activity**									
Case (*n* (%))	181	(56.4)	299	(53.1)	98	(59.8)	43	(71.7)	
Model 1 ^c^	1.00	(Ref)	0.87	(0.65 to 1.15)	1.19	(0.80 to 1.77)	1.73	(0.93 to 3.22)	0.060
Model 2 ^d^	1.00	(Ref)	0.85	(0.62 to 1.15)	1.21	(0.78 to 1.87)	1.64	(0.84 to 3.21)	0.180
Model 3 ^e^	1.00	(Ref)	0.79	(0.58 to 1.08)	1.10	(0.70 to 1.70)	1.38	(0.70 to 2.72)	0.483
**Weight loss**									
Case (*n* (%))	38	(11.8)	87	(15.5)	28	(17.1)	15	(25.0)	
Model 1 ^c^	1.00	(Ref)	1.30	(0.85 to 1.97)	1.41	(0.80 to 2.48)	1.98	(0.94 to 4.16)	0.127
Model 2 ^d^	1.00	(Ref)	1.20	(0.78 to 1.84)	1.33	(0.75 to 2.37)	1.85	(0.85 to 3.99)	0.114
Model 3 ^e^	1.00	(Ref)	1.20	(0.78 to 1.84)	1.34	(0.75 to 2.39)	1.82	(0.83 to 4.02)	0.128

Abbreviation: ^a^ All values were counts (%) or relative odds ratio (95% confidence interval). All estimates are derived from multivariable logistic regression models; ^b^ Linear trend *p*-values are calculated with the likelihood ratio test using ordinal variable (color-changing chewing gum score) values. Bolded *p*-values are statistically significant (*p* < 0.05); ^c^ Model 1 was adjusted for age (continuous), sex (woman or man), and population density (≥1000 or <1000 people/km^2^); ^d^ Model 2 included variables from model 1 as well as adjustment for BMI (continuous), physical activity (yes or no), smoking status (never smoker, past smoker, or current smoker), alcohol status (drinker or nondrinker), educational attainment (<9, 10–12, or ≥13 years), medications prescribed (continuous), living alone (yes or no), socioeconomic status (high or low), and history of disease (hypertension, diabetes, dyslipidemia, heart disease, and stroke; yes or no); ^e^ Model 3 included variables from model 2 plus adjustment for denture use (yes or no) and daily teeth brushing (yes or no).

**Table 4:** Multivariable adjusted odds ratios and 95% confidence intervals of the prevalence of each component of the KCL according to the score of color-changing chewing gum ^a^.

	Color-Changing Chewing Gum Score								*p* for Trend ^b^
	5 (*n* = 320)		4 (*n* = 563)		3 (*n* = 163)		1 + 2 (*n* = 60)		
**IADL disability**									
Case (*n* (%))	8	(2.5)	34	(6.0)	7	(4.3)	4	(6.7)	
Model 1 ^c^	1.00	(Ref)	2.22	(1.00 to 4.96)	0.94	(0.30 to 2.99)	1.30	(0.32 to 5.27)	0.986
Model 2 ^d^	1.00	(Ref)	2.35	(1.02 to 5.40)	0.84	(0.25 to 2.82)	1.23	(0.28 to 5.49)	0.900
Model 3 ^e^	1.00	(Ref)	2.35	(1.02 to 5.44)	0.84	(0.25 to 2.85)	1.25	(0.27 to 5.80)	0.898
**Physical**									
Case (*n* (%))	42	(13.1)	90	(16.0)	32	(19.5)	8	(13.3)	
Model 1 ^c^	1.00	(Ref)	1.19	(0.78 to 1.81)	1.19	(0.69 to 2.05)	0.66	(0.28 to 1.57)	0.787
Model 2 ^d^	1.00	(Ref)	1.20	(0.77 to 1.87)	1.15	(0.65 to 2.03)	0.63	(0.25 to 1.56)	0.711
Model 3 ^e^	1.00	(Ref)	1.20	(0.77 to 1.87)	1.19	(0.67 to 2.11)	0.61	(0.24 to 1.55)	0.729
**Nutrition**									
Case (*n* (%))	2	(0.6)	13	(2.3)	2	(1.2)	4	(6.7)	
Model 1 ^c^	1.00	(Ref)	3.85	(0.85 to 17.32)	1.71	(0.23 to 12.50)	6.03	(0.94 to 38.59)	0.174
Model 2 ^d^	1.00	(Ref)	5.50	(0.94 to 32.29)	1.80	(0.19 to 17.56)	3.18	(0.35 to 28.61)	0.663
Model 3 ^e^	1.00	(Ref)	6.72	(1.06 to 42.82)	2.94	(0.27 to 31.73)	4.06	(0.39 to 41.73)	0.486
**Oral**									
Case (*n* (%))	56	(17.5)	116	(20.6)	50	(30.5)	26	(43.3)	
Model 1 ^c^	1.00	(Ref)	1.23	(0.85 to 1.76)	1.80	(1.13 to 2.86)	2.91	(1.59 to 5.35)	**<0.001**
Model 2 ^d^	1.00	(Ref)	1.17	(0.81 to 1.71)	1.69	(1.05 to 2.72)	2.56	(1.36 to 4.82)	**0.001**
Model 3 ^e^	1.00	(Ref)	1.09	(0.74 to 1.59)	1.47	(0.91 to 2.398)	2.20	(1.15 to 4.19)	**0.011**
**Social**									
Case (*n* (%))	11	(3.4)	28	(5.0)	15	(9.2)	5	(8.3)	
Model 1 ^c^	1.00	(Ref)	1.37	(0.67 to 2.82)	2.19	(0.95 to 5.06)	1.80	(0.58 to 5.60)	0.094
Model 2 ^d^	1.00	(Ref)	1.37	(0.65 to 2.88)	2.11	(0.88 to 5.03)	1.69	(0.52 to 5.51)	0.138
Model 3 ^e^	1.00	(Ref)	1.32	(0.62 to 2.80)	2.02	(0.83 to 4.89)	1.51	(0.45 to 5.10)	0.202
**Cognitive**									
Case (*n* (%))	104	(32.4)	183	(32.5)	53	(32.3)	17	(28.3)	
Model 1 ^c^	1.00	(Ref)	1.03	(0.76 to 1.38)	0.96	(0.63 to 1.46)	0.73	(0.39 to 1.36)	0.465
Model 2 ^d^	1.00	(Ref)	1.01	(0.74 to 1.37)	0.89	(0.58 to 1.36)	0.66	(0.35 to 1.24)	0.252
Model 3 ^e^	1.00	(Ref)	0.99	(0.72 to 1.34)	0.86	(0.56 to 1.33)	0.62	(0.32 to 1.18)	0.183
**Depression**									
Case (*n* (%))	66	(20.6)	123	(21.9)	52	(31.7)	22	(36.7)	
Model 1 ^c^	1.00	(Ref)	1.07	(0.76 to 1.51)	1.57	(1.00 to 2.45)	1.69	(1.12 to 3.15)	**0.004**
Model 2 ^d^	1.00	(Ref)	1.06	(0.74 to 1.51)	1.48	(0.93 to 2.35)	1.59	(1.05 to 2.90)	**0.019**
Model 3 ^e^	1.00	(Ref)	1.02	(0.71 to 1.47)	1.45	(0.91 to 2.32)	1.58	(1.04 to 2.90)	**0.024**

Abbreviations: IADL = instrumental activities of daily living; KCL = Kihon Checklist; ^a^ All values were counts (%) or relative odds ratio (95% confidence interval). All estimates are derived from multivariable logistic regression models; ^b^ Linear trend *p*-values are calculated with the likelihood ratio test using ordinal variable (color-changing chewing gum score) values. Bolded *p*-values are statistically significant (*p* < 0.05); ^c^ Model 1 was adjusted for age (continuous), sex (woman or man), and population density (≥1000 or <1000 people/km^2^); ^d^ Model 2 included variables from model 1 as well as adjustment for BMI (continuous), physical activity (yes or no), smoking status (never smoker, past smoker, or current smoker), alcohol status (drinker or nondrinker), educational attainment (<9, 10–12, or ≥13 years), medications prescribed (continuous), living alone (yes or no), socioeconomic status (high or low), and history of disease (hypertension, diabetes, dyslipidemia, heart disease, and stroke; yes or no); ^e^ Model 3 included variables from model 2 plus adjustment for denture use (yes or no) and daily teeth brushing (yes or no).
